# The plasticity of *Plasmodium falciparum *gametocytaemia in relation to age in Burkina Faso

**DOI:** 10.1186/1475-2875-9-281

**Published:** 2010-10-12

**Authors:** André Lin Ouédraogo, Teun Bousema, Sake J de Vlas, Nadine Cuzin-Ouattara, Jan-Peter Verhave, Chris Drakeley, Adrian JF Luty, Robert Sauerwein

**Affiliations:** 1 Centre National de Recherche et de Formation sur le Paludisme, BP 2208, Ouagadougou 01, Burkina Faso; 2 Department of Medical Microbiology, Radboud University Nijmegen Medical Centre, Nijmegen, The Netherlands; 3 Department of Infectious & Tropical Diseases, London School of Hygiene & Tropical Medicine, London, UK; 4 Department of Public Health, Erasmus MC, University Medical Center Rotterdam, P.O. Box 2040, 3000 CA Rotterdam, The Netherlands

## Abstract

**Background:**

Malaria transmission depends on the presence of gametocytes in the peripheral blood. In this study, the age-dependency of gametocytaemia was examined by microscopy and molecular tools.

**Methods:**

A total of 5,383 blood samples from individuals of all ages were collected over six cross sectional surveys in Burkina Faso. One cross-sectional study used quantitative nucleic acid sequence based amplification (QT-NASBA) for parasite quantification (n = 412). The proportion of infections with concurrent gametocytaemia and median proportion of gametocytes among all parasites were calculated.

**Results:**

Asexual parasite prevalence and gametocyte prevalence decreased with age. Gametocytes made up 1.8% of the total parasite population detected by microscopy in the youngest age group. This proportion gradually increased to 18.2% in adults (p < 0.001). Similarly, gametocytes made up 0.2% of the total parasite population detected by QT-NASBA in the youngest age group, increasing to 5.7% in adults (p < 0.001). This age pattern in gametocytaemia was also evident in the proportion of gametocyte positive slides without concomitant asexual parasites which increased from 13.4% (17/127) in children to 45.6% (52/114) in adults (OR 1.55, 95% CI 1.38-1.74, p < 0.001).

**Conclusions:**

The findings of this study suggest that although gametocytes are most commonly detected in children, the proportion of asexual parasites that is committed to develop into gametocytes may increase with age. These findings underscore the importance of adults for the human infectious reservoir for malaria.

## Background

The malaria parasite's life- cycle is composed of several developmental stages, one of which is the transmissible sexual stage comprising male and female gametocytes. Mature gametocytes are apparently benign, causing no overt disease symptoms. They appear to be developmentally arrested at the G_0 _phase of the cell cycle and circulate within erythrocytes in the peripheral blood of the human host until they are taken up by a feeding female mosquito. In the mosquito midgut, gametocyte activation and fertilization take place. The subsequent formation of sporogonic stages results in the development of thousands of sporozoites that migrate to and invade the salivary glands, rendering the mosquito infectious to humans.

During the course of an infection with *Plasmodium falciparum*, gametocytes are generated from asexual stage parasites. Only a small fraction of the asexual parasites of *P. falciparum *commit to form gametocytes [1] and as a result only a fraction of infected individuals also harbour gametocytes [1,2]. It is now understood that this apparently low occurrence of gametocytes is partly a reflection of the low sensitivity of microscopy for the detection of gametocytes [2,3]. However, the fact remains that asexual parasitaemia is not always accompanied by gametocyte carriage [1-3], and that the relationship between asexual parasite density and gametocyte prevalence or density is not straightforward. Some studies report a positive association between asexual parasite densities and gametocyte prevalence [4-6] and density [4] while others observe inverse associations [7,8] or report that the association may be modified by age [2].

Factors that trigger and regulate the commitment of asexual stage parasites to gametocytes are largely unknown but are thought to include intrinsic parasite factors [9], anti-malarial treatment [4,6,10] and treatment outcome [4-6,11] , fever [7,8], haematological disruptions [6,12,13] and the presence of competing parasite strains [14,15] or species [6,16]. In general the mechanism of sexual commitment appears to be highly plastic and environment sensitive [17,18]. The flexible gametocyte production can be interpreted as a response mechanism of the parasite to stressful situations: if the survival of the asexual stage parasite is challenged, the investment in transmission stages increases.

Here, we explore age-dependent variation in gametocytaemia in a series of cross-sectional surveys in an area of seasonal malaria transmission in Burkina Faso, using both microscopy and quantitative nucleic acid sequence based amplification (QT-NASBA).

## Methods

### Study site and population

The study was carried out in the vicinity of Ouagadougou, the capital of Burkina Faso. The area has the ecological characteristics of Sudan savannah. Participating populations from six villages (longitude: 1°46'- 1°79'; latitude: 12°52'-12°61') were of the same ethnic group (Mossi) and had similar age distributions. Transmission intensity is intense and seasonal in this region. Study subjects were given detailed explanations of the procedures, risk and benefits involved in the study and their consent was obtained. The study protocol was viewed and approved by the Ministry of Health of Burkina Faso (Research's Authorization number 2000/3174/MS/SG/DEP).

### Blood sample collection

Cross-sectional surveys were performed in January, May, August and December 2002 and in April and December 2003. Participants were randomly selected from previously determined age groups (0.5-4, 5-9, 10-14, 15-24 and 25+ years) based on census lists and computer generated randomization tables. Thick and thin blood smears were made from finger-prick blood. The body temperature was measured and febrile individuals who were parasitaemic were treated with chloroquine according to the national policy in 2002. In the cross-sectional survey of December 2003, a single finger prick sample was used for blood smears and the collection of nucleic acids for quantitative-nucleic acid sequence based amplification (QT-NASBA); 100 μL blood samples were collected from 412 volunteers of all ages from the six villages. The first part of the RNA extraction was done in the field following the original guanidinium isothiocyanate (GuSCN) RNA extraction method [19] until the nucleic acids were bound to silica dioxide particles. At this point, samples were stored at -20°C and transferred to the laboratory for completion of the extraction and QT-NASBA analysis.

### Microscopical detection of *P. falciparum *parasites

Samples were considered negative if no parasites were detected in 100 high-power fields of Giemsa-stained thick blood smears. Both asexual stage and gametocyte densities were assessed in the thick smear by counting against 500 and 1,000 leucocytes, respectively. Based on this approach, the lower limit of microscopy for gametocyte quantification was estimated at 8 gametocytes/μl of blood. Parasite counts were converted to numbers of parasites per μl by assuming a standard count of 8,000 leucocytes/μl of blood. Each sample was read independently by two microscopists and the mean density was used. A third reader was involved when the first two readers disagreed about the prevalence of gametocytes or their estimated densities differed ≥30%. In these cases the mean density of the two closest readings was used.

### Real-time Pfs25 QT-NASBA and nucleic acid extraction

18S rRNA real-time QT-NASBA and Pfs25 real time mRNA QT-NASBA were performed as described elsewhere [20]. Briefly, real-time QT-NASBA for *Pfs*25 mRNA (Genbank accession number AF193769.1) was performed on a Nuclisens EasyQ analyser (bioMérieux) using the Nuclisens Basic Kit for amplification according to manufacturer's instructions at a KCl concentration of 80 mM. Reactions were performed in a total reaction volume of 10 μl per reaction. For quantification, time to positivity is calculated, i.e. the time point during amplification at which the fluorescence detecting target amplicons becomes higher than the mean fluorescence of three negative controls + 20 standard deviations (SD). The use of a standard gametocyte stage V dilution series allows exact calculation of the number of gametocytes present in unknown samples [20]. The sensitivity of this method is 20-100 gametocytes/mL.

### Statistical analysis

The age-dependency of gametocytaemia by microscopy was determined by categorizing the population into 0.5-2, 3-4, 5-9, 10-14, 15-19 and ≥20 year-old individuals [2]. For QT-NASBA data, numbers were smaller and age groups were combined to form 0.5-4, 5-9, 10-19 and ≥20 year-old individuals. The proportion of asexual stage parasite carriers that also harboured gametocytes was calculated. Similarly, the individual proportion of gametocytes among total parasites was calculated by dividing the individual gametocyte density by the total parasite density (asexual parasite density plus gametocyte density). An identical approach was used for QT-NASBA where the *Pfs*25 QT-NASBA gametocyte prevalence or density was divided by the 18S QT-NASBA total parasite prevalence or density. Age-dependent trends in parasite carriage were determined by linear (density after log transformation) or logistic (prevalence) regression models and regression coefficients or odds ratios are presented, respectively. Estimates were adjusted for seasonality (dry, start wet, peak wet and end wet) where appropriate. The majority of individuals were only included in one of the surveys. Several individuals were included in more than one cross-sectional survey with a time-gap between repeated measures of two months (n = 484) or ≥4 months (n = 781). Adjusting for the correlation between observations from the same individual by generalized estimating equations (GEE) did not indicate a significant impact of autocorrelation (i.e. estimates and confidence intervals remained unaltered) and, therefore, conventional regression models were used. Trends in other variables were determined by χ^2^-test for trend or non-parametric trend tests for continuous variables. All statistical analyses were performed using STATA 11 [Statacorp, Texas US].

## Results

Overall, the six cross-sectional studies yielded a total of 5,383 observations: 1,216 observations from January 2002, 968 from June, 749 from August, 599 from December, 1,084 from April 2003 and 767 from December 2003. 58.1% of the participants were of the female gender (3,127/5,383). Details on the seasonality of parasite carriage were presented previously [21]. These findings were derived from 3154 individuals, 59.9% (1889/3154) of whom were seen once, 21.7% (685/3154) twice, 10.1% (320/3154) three times, 5.1% (161/3154) four times, 2.4% (75/3154) five times and 0.8% (24/3154) six times. The vast majority of individuals who donated a blood sample were afebrile: Only 5.1% of children below 15 years of age had a temperature ≥37.5°C (165/3,250) and 1.0% of older individuals (21/2,105).

**Figure 1:**
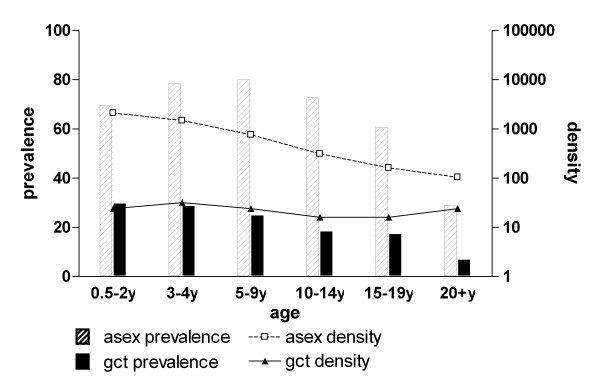
**Parasite carriage in different age groups by microscopy**. Asex = asexual parasite; gcyt = gametocyte. The number of asexual parasite carriers (with gametocytes) for the different age groups was 0.5-2y: 297 (127); 3-4y: 468 (171); 5-9y: 1053(326); 10-14y: 690(174); 15-19y: 254(72); 20 + y: 285(114).

**Figure 2:**
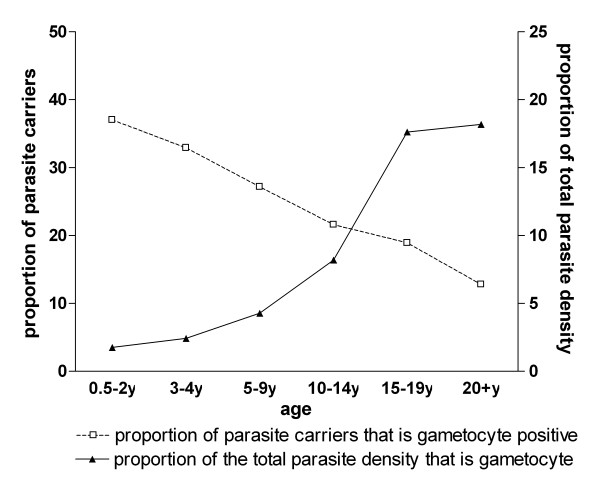
**The prevalence and density of gametocytes relative to total parasite carriage by microscopy**. The number of asexual parasite carriers (with gametocytes) for the different age groups was 0.5-2y: 297 (127); 3-4y: 468 (171); 5-9y: 1053(326); 10-14y: 690(174); 15-19y: 254(72); 20+y: 285(114). Note: data for those carrying gametocytes in the absence of asexual parasitaemia were excluded (see Figure 3).

**Figure 3:**
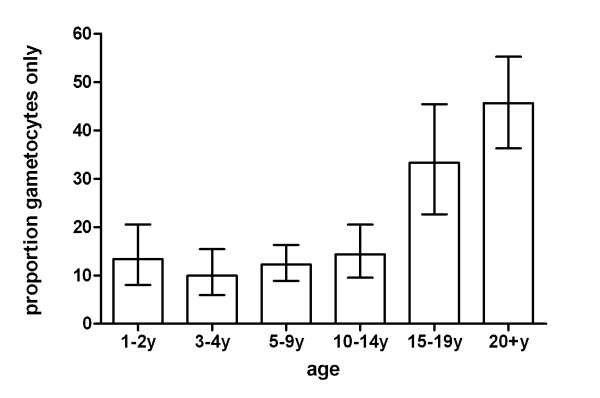
**Gametocyte carriage in the absence of microscopically detectable asexual parasites**. Bars indicate the proportion of microscopically detected gametocyte carriers in each age category without microscopically confirmed asexual parasites. Error bars indicate the upper and lower limit of the 95% confidence interval around the proportion. The number of gametocyte carriers (without concurrent asexual parasitaemia) for the different age groups was 0.5-2y: 127(17); 2-3y: 171(17); 5-9y: 326(40); 10-15y 174(25); 15-20y: 72(24); 20+y: 114(52).

The prevalence of asexual parasites (OR 0.59; 95% CI 0.57-0.62, p < 0.001) and gametocytes (OR 0.70; 95% CI 0.67-0.74, p < 0.001) decreased with age (Figure 1). In parasite positive individuals, the log-transformed density of asexual parasites also decreased over age categories (β = -0.23; 95% CI -0.24 - -0.21, p < 0.001). The gametocyte density in gametocyte carriers also decreased significantly but to a lesser extent (β = -0.041; 95% CI -0.055 - -0.028, p < 0.001). The proportion of infections with concomitant gametocytaemia decreased with age (Figure 2). Whilst 37.0% (110/297) of 0.5-2 year-old carriers of asexual stage parasites concurrently had gametocytes, this was only true for 12.8% (62/485) of the parasite carriers who were 20 years old and above (OR = 0.76;95% CI 0.72-0.81, p < 0.001). In contrast with this trend, the median proportion of gametocytes among all parasites increased with increasing age (Figure 2). Thus, whilst gametocytes only represented 1.8% of the density of the total parasite population in the youngest age group, this proportion gradually increased to a peak of 18.2% in adults (β = 0.42; 95% CI 0.33-0.51, p < 0.001). Although the majority of gametocyte carriers also harboured asexual parasites, microscopical evidence of concurrent asexual parasitaemia was lacking in 17.8% (175/984) of all gametocyte carriers. The proportion of gametocyte carriers without concurrent asexual parasites detected by microscopy was 13.4% (17/127) in children below two years of age and increased with age to 45.6% (52/114) in the oldest age group (Figure 3; trend for age in categories OR 1.55, 95% CI 1.38-1.74, p < 0.001).

**Figure 4:**
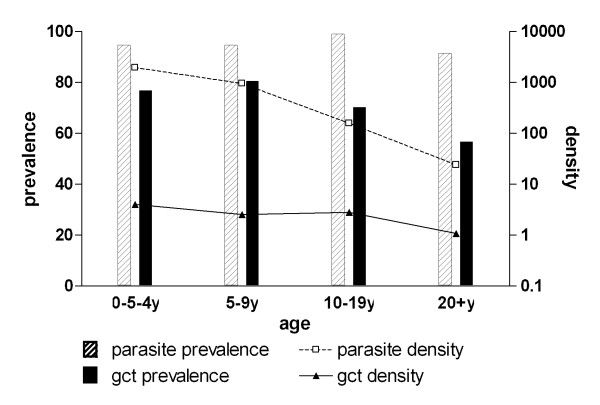
**Parasite carriage by quantitative nucleic acid based amplification (QT-NASBA) in different age groups**. Total parasite prevalence and density were determined by 18S QT-NASBA; gametocyte prevalence and density by *Pfs*25 QT-NASBA. The number of parasite carriers (with concurrent gametocytaemia) for the different age groups was 0.5-4y: 74(60); 5-9y: 93(79); 10-19y: 117(83); 20 + y 108(67).

**Figure 5:**
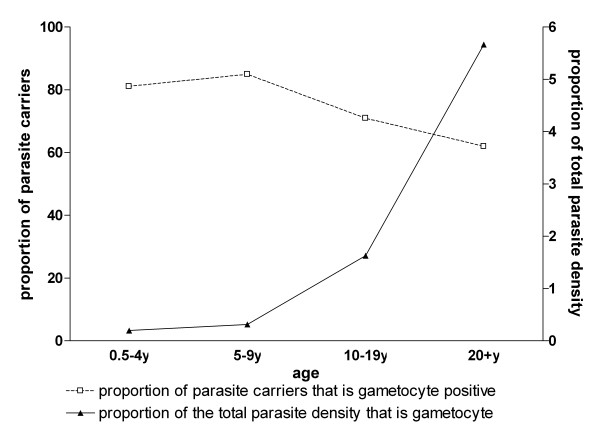
**The age-dependent prevalence and density of gametocytes relative to total parasite carriage by QT-NASBA**. The number of parasite carriers (with concurrent gametocytaemia) for the different age groups was 0.5-4y: 74(60); 5-9y: 93(79); 10-19y: 117(83); 20+y 108(67).

Patterns of gametocyte carriage and asexual parasite carriage were unaffected by the presence of fever and did not change when molecular methods were used for parasite detection instead of microscopy. For the limited number of samples (n = 412) for which both 18S QT-NASBA (total parasite) and *Pfs25 *QT-NASBA (gametocyte) prevalence and density data were available, total parasite prevalence (OR = 0.85; 95% CI 0.63-1.15, p = 0.29) and gametocyte prevalence (OR = 0.73; 95% CI 0.63-0.85, p < 0.001) both decreased with increasing age (Figure 4). Log-transformed total parasite density showed a decrease with age (β = -0.36; 95% CI -0.44 - -0.28, p < 0.001) that was more pronounced than that of gametocyte density (β = -0.096; 95% CI -0.17 - -0.017, p = 0.02). Densities of gametocytes detected by *Pfs25 *QT-NASBA in gametocyte carriers were low with a median gametocyte density of 2.3 gametocytes/μL (IQR 0.4-10). Similar to observations by microscopy, the proportion of parasite carriers with concurrent gametocytes decreased with age. While 81.1% (60/74) of parasite carriers in the youngest age group harboured gametocytes, this proportion gradually decreased to 62.0% (67/108) in the oldest age group (OR = 0.75;95% CI 0.64-0.87, p < 0.001). On the other hand, and again consistent with the pattern revealed by microscopy, the median proportion of gametocytes among total parasites increased from 0.2% in the youngest to 5.7% in the oldest age group (β = 0.61; 95% CI 0.35-0.86, p < 0.001, Figure 5).

## Discussion

This study indicates that, while the global prevalence of asexual parasites and gametocytes is highest in children, the density of gametocytes relative to the total parasite concentration increases with age. This age-dependent increase is apparent by microscopy and by a more sensitive molecular gametocyte detection technique. These findings suggest that the commitment of asexual malaria parasites to the sexual pathway may increase with age.

*Plasmodium falciparum *sexual stage commitment is crucial for the transmission of parasites from man to mosquito and the subsequent spread of malaria in the human population. A better understanding of the factors that influence the switch from asexual to sexual stage development in the human host would therefore provide new opportunities for malaria control. In the study presented here, a large data set collected during cross sectional surveys in two consecutive years was analysed to explore factors associated with gametocyte production. Numerous studies have shown that the prevalence of gametocytes decreases with age [4,5,22-28]. The present study attempted to determine whether this is the simple consequence of an age-dependent decline in the prevalence and density of asexual parasites [4-6] or if there may be an age-dependent production of gametocytes. The proportion of gametocytes relative to total parasite density increased with age in the current study [2]. Whilst gametocytes only comprised 1.8% of the total parasite population in the youngest children, they comprised 18.2% of the total parasite population in the oldest age-group. These calculations may have been affected by the limited sensitivity of microscopy for detecting low gametocyte densities [29], but the association between age and gametocyte production was supported by two additional lines of evidence.

In the analyses on the proportion of asexual parasite carriers with concurrent gametocytaemia and the proportion of gametocytes among the total parasite population, the group of individuals with microscopically detectable gametocytes but no asexual parasites was excluded. This group was expected to form a relatively small subset of individuals whose asexual parasites were recently cleared by immune responses or anti-malarial drugs. Contrary to this expectation, the presence of gametocytes in the absence of asexual parasites detectable by microscopy was not rare: one in six gametocyte carriers had no microscopical evidence of asexual parasitaemia and this proportion increased with age. One theoretical explanation for this observation could be that a higher proportion of infections were recently cleared by anti-malarial drugs in adults. After the clearance of asexual parasites by chloroquine, the most commonly used anti-malarial drug in the study area, gametocytes may have persisted since these are largely unaffected by this drug [30]. No information on recent drug use was recorded in this study but it is unlikely that a difference in drug use between children and adults would explain the observations. Disease symptoms and anti-malarial drug use are more common in children, not in adults [31]. A more likely explanation is that that low density (i.e. microscopically undetectable) malaria infections were more frequently accompanied by microscopically detectable gametocyte densities in older age-groups. This would support an age-dependent increase in commitment to gametocyte production.

A last line of evidence comes from the molecular gametocyte detection tool that is at least 100-fold more sensitive for detection of gametocytes [32]. QT-NASBA detects gametocytes at densities as low as 0.02 gametocytes/μL [2,20]. Similar to microscopical findings, the proportion of gametocytes relative to total parasite density detectable by QT-NASBA increased with age: from 0.2% in the youngest age group to 5.7% in the oldest individuals. These three lines of evidence suggest that the relative density of gametocytes increases with age in an area where transmission is intense and adults have developed an efficient anti-disease and anti-parasite immune response [33,34].

The biological mechanism behind an age-dependent increase in sexual stage commitment is impossible to deduce from epidemiological data. It could plausibly result from epidemiological differences in infections between age-groups or from a response of the parasite to age-acquired immune responses and the resulting lower density of asexual parasites. The latter would suggest a strategic advantage for parasites that may increase gametocytaemia in response to conditions that negatively affect asexual stage parasite multiplication [35]. The developmental decision to enter gametocytogenesis for *P. falciparum *occurs during the formation of the asexual schizont, which can commit its entire progeny of merozoites either to develop once more asexually, or to enter sexual differentiation [36]. This decision may depend on the immune stress experienced by the parasites. Gametocytogenesis in *Plasmodium chabaudi *is increased in immunized compared to naïve mice [37]. Similar findings for *P. falciparum *have been reported when parasites were exposed to immune stimuli *in vitro *[38]. The findings from the current study suggest a similar mechanism in natural infections where gametocytaemia increases in response to age-acquired immune responses, decreasing asexual parasite densities or other age-dependent factors. Anaemia and drug treatment, likely triggers of gametocyte production [6,12,13] were not directly measured but are likely to be more common in children than adults and can therefore not explain the findings of a higher relative gametocyte density in adults. It is also possible that the association is explained by a longer duration of malaria infections in adults. Children are more likely to develop symptoms and seek treatment or benefit from a prophylactic effect of presumptive treatment [39]. Infections may have a longer average duration in immune adults, giving parasites more time to develop gametocytes. The half-life of gametocytes is 3-6 days [40,41] and the density of gametocytes could therefore increase cumulatively when gametocytes have been produced for a longer period of time while asexual parasite densities are decreasing prior to sampling. Such an effect cannot be ruled out in this study although the asymptomatic nature of the vast majority of infections makes it plausible that most individuals harboured parasites for a sufficiently long time to develop gametocytes [41]. It is also possible that gametocyte mortality is increased in children due to the high asexual parasite density-mediated release of cytokines [42,43], resulting in a lower relative gametocyte density. Gametocyte-clearing immune responses [44] may also have contributed to these observations but there is currently insufficient evidence for the functional relevance of such responses in malaria endemic countries or its age-dependency.

## Conclusions

In conclusion, the findings reported in this study suggest that once asexual population growth has been controlled by the host, the transmission benefits of increased gametocyte densities become apparent. These findings require confirmation in longitudinal studies that should ideally use molecular parasite detection tools. Although the patterns observed by QT-NASBA were similar to those by microscopy, the former is preferable since it detects gametocytes over a much wider range of densities [20]. An additional advantage of molecular detection tools is that they would also allow the detection of sexual stage committed asexual parasites [45], which could provide an additional level of detail in studying the dynamics of gametocyte production. These findings have an important implication for malaria control: although the prevalence of asexual parasite and gametocyte carriage decreases with increasing age, adults can be important contributors to the human infectious reservoir. Adults constitute the majority of populations in malaria endemic areas and many adults harbour (low densities of gametocytes. The current study suggests that the commitment to gametocytaemia may increase in adults, increasing their relative importance for the human infectious reservoir. Adults should, therefore, be taken into consideration when implementing interventions that aim at reducing malaria transmission.

## Competing interests

The authors declare that they have no competing interests.

## Authors' contributions

ALO and TB analysed the data and wrote the manuscript; ALO, NCO, SV, JPV and RS were responsible for the original study design and data collection; SV, CD and AJFL contributed in data analysis; CD, SV, AJFL and RS contributed to data interpretation and manuscript preparation. All authors read and approved the final manuscript.
